# Supplementary material for: The effects of prenatal dietary supplements on blood glucose and lipid metabolism in gestational diabetes mellitus patients: A systematic review and network meta-analysis protocol of randomized controlled trials
Source: PLoS One. 2022 May 3;17(5):e0267854. doi: 10.1371/journal.pone.0267854 (PMC9064104; doi:10.1371/journal.pone.0267854)
Supplement: S2 File — (PDF) [file pone.0267854.s002.pdf]

Proposed data abstraction form

Intervention review - Randomized controlled trials

Title: The effects of prenatal dietary supplements on blood glucose and lipid metabolism in gestational diabetes mellitus patients: A systematic review and network meta-analysis protocol of randomized controlled trials

Authors: Dr. Sumanta Saha and Dr. Sujata Saha

Please fill out this form for each of the studies included in this review.

* Required

Reviewer author's detail

1. Name

____

2. Date of data abstraction

____

Example: January 7, 2019

3. Email id

____

Is the study population eligible for inclusion in this review?

4. What is the diagnosis of trial participants?

Mark only one oval.

- ( ) Gestational diabetes melitus
- ( ) Other: _______________________

Study design

5. Is the study design eligible for inclusion in this review? *

The eligible study design will be a parallel-arm randomized controlled trial with participants of an intervention arm not receiving the interventions of interest but may receive a placebo.

Mark only one oval.

- Yes
- No
- Other: ____________________

Study details

Please enter the details of the respective studies.

6. Last name of the first author of the publication

____

7. Year of publication

____

8. Digital object identifier (if avaialble)

____

9. PMID (if available)

____

10. Web link

If a PMID or DOI is unavailable.

____

11. Enlist the country or countries where the trial got conducted.

12. Trial registration no.

13. The trial is

Mark only one oval.

- Single centered
- Multicentered
- Other:

14. Trial duration

15. Funding information available in the article

Mark only one oval.

- Yes
- No
- Other:

16. Ethical clearance for the study obtained

Mark only one oval.

- Yes
- No
- Other: ____________________

17. Participant consent obtained

Mark only one oval.

- Yes
- No
- Other: ____________________

18. Diagnostic criteria used to diagnosis gestational diabetes mellitus

Mark only one oval.

- American Diabetes Association
- International Association of Diabetes and Pregnancy Study Groups criteria
- Carpenter and Coustan’s criteria
- European Diabetic Pregnancy Study Group
- Other: ____________________

19. At which gestational age were the participants recruited in the trial?

20. Did the study population include women with a previous history of gestational diabetes mellitus?

Mark only one oval.

- Yes
- No
- Unclear
- Other: ____________________

Data of dietary supplement receiving arm/s

21. No. of arms

____

Arm 1

22. Intervention received

____

23. Intervention dosage and regimen

____

24. How many participants were randomized to this arm?

____

25. Mean age (years, standar deviation)

Arm 2

Skip if one intervention arm received nutrional supplementation

26. Intervention received

27. Intervention dosage and regimen

28. How many participants were randomized to this intervention arm?

29. Mean age (years, standar deviation)

Arm 3 onwards

Use this section if three or more treatment arms received nutritional supplement (enter detail of respective treatment arms in separate lines)

30. Intervention received

31. Intervention dosage and regimen

32. How many participants were randomized to this arm?

33. Mean age (years, standar deviation)

Data of placebo or no intervention receiving arm

34. Intervention received

35. Intervention dosage and regimen (where available)

36. How many participants were randomized to this arm?

37. Mean age (years, standar deviation)

Outcome data

38. Select (tick) outcomes reported in this study. *

Check all that apply.

	Reported	Not reported
Fasting plasma glucose	☐	☐
Glycated hemoglobin	☐	☐
Homeostasis model assessment of insulin resistance (HOMA-IR)	☐	☐
Homeostasis model assessment of β-cell function (HOMA-B)	☐	☐
Quantitative insulin sensitivity check index (QUICKI)	☐	☐
High-density lipoprotein (HDL) cholesterol	☐	☐
Low-density lipoprotein (LDL) cholesterol	☐	☐
Very-low-density lipoprotein (VLDL) cholesterol	☐	☐
Total cholesterol	☐	☐
Triglycerides	☐	☐
Triglyceride to HDL ratio	☐	☐

This content is neither created nor endorsed by Google.

Google Forms
