# Supplementary material for: The effects of prenatal dietary supplements on blood glucose and lipid metabolism in gestational diabetes mellitus patients: A systematic review and network meta-analysis protocol of randomized controlled trials
Source: PLoS One. 2022 May 3;17(5):e0267854. doi: 10.1371/journal.pone.0267854 (PMC9064104; doi:10.1371/journal.pone.0267854)
Supplement: S3 File — (PDF) [file pone.0267854.s003.pdf]

Data abstraction form for meta-analysis (Draft copy)

Title: The effects of prenatal dietary supplements on blood glucose and lipid metabolism in gestational diabetes mellitus patients: A systematic review and network meta-analysis protocol of randomized controlled trials

Prepared by Dr. Sumanta Saha and Dr. Sujata Saha

Instruction to fill this form: Leave no cell empty. If data is unavailable for a cell, enter a hyphen (-) or mention 'NA' (not applicable) in that cell.

First author’s last name	Year of publication	Title of the study	Outcome	Intervention given in the treatment arm (e.g., vitamin D, no intervention, placebo)	Unit (as reported in the trial; without conversion)	End point mean	Standard deviation (SD) of endpoint mean	Total participants (included in the analysis)	If endpoint mean/SD is unavailable, enter data as reported in the trial	DOI	PMID
